# A single dose of trichloroethylene given during development does not substantially alter markers of neuroinflammation in brains of adult mice

**DOI:** 10.1080/1547691X.2017.1305021

**Published:** 2017-12

**Authors:** Jacqueline R. Meadows, Chevonne Parker, Kathleen M. Gilbert, Sarah J. Blossom, Jamie C. DeWitt

**Affiliations:** a Department of Pharmacology and Toxicology, Brody School of Medicine, East Carolina University Greenville, NC, USA; b Department of Microbiology and Immunology, UAMS College of Medicine, Arkansas Children’s Research Institute, Little Rock, AR, USA; c Department of Pediatrics, UAMS College of Medicine, Arkansas Children’s Research Institute, Little Rock, AR, USA

**Keywords:** Trichloroethylene, neuroinflammation, developmental, early-life, microglia, brain

## Abstract

Trichloroethylene (TCE) is a widespread environmental contaminant associated with developmental immunotoxicity and neurotoxicity. Previous studies have shown that MRL^+/+^ mice exposed to TCE from gestation through early-life demonstrate robust increases in inflammatory markers in peripheral CD4^+^ T-cells, as well as glutathione depletion and increased oxidative stress in cerebellum-associated with alterations in behavior. Since increased oxidative stress is associated with neuroinflammation, we hypothesized that neuroinflammatory markers could be altered relative to unexposed mice. MRL^+/+^ mice were given 0.5 mg/ml of TCE in vehicle or vehicle (water with 1% Alkamuls EL-620) from conception through early adulthood via drinking water to dams and then directly to post-weaning offspring. Animals were euthanized at 49 days of age and levels of pro- and anti-inflammatory cytokines, density of T-cell staining, and micro-glial morphology were evaluated in brains to begin to ascertain a neuroinflammatory profile. Levels of IL-6 were decreased in female animals and while not statistically significant, and levels of IL-10 were higher in brains of exposed male and female animals. Supportive of this observation, although not statistically significant, the number of ameboid microglia was higher in exposed relative to unexposed animals. This overall profile suggests the emergence of an anti-inflammatory/neuroprotective phenotype in exposed animals, possibly as a compensatory response to neuroinflammation that is known to be induced by developmental exposure to TCE.

## Introduction

Trichloroethylene (TCE) is an organic solvent most commonly used as a degreasing agent in myriad industrial settings. Its widespread industrial use and inappropriate disposal over the years has putatively resulted in its prevalence as a widespread environmental contaminant, notably in surface and groundwater. Based on the likelihood of exposure together with negative health impacts, TCE is consistently ranked 16th out of 275 on the CERCLA list of hazardous chemicals. As noted in the 2011 US Environmental Protection Agency (USEPA) toxicological review, one of the most sensitive noncancer outcomes associated with TCE exposure in humans is immunotoxicity, and the spectrum of TCE-related immune dysfunctions including inflammation has the potential to produce adverse effects in the brain (USEPA 2011).

Aside from immunotoxic effects of TCE on T-cells observed in both human and mouse studies (Byers et al. 1988; Peden-Adams et al. 2006; Blossom & Doss 2007; Blossom et al. 2008; Gilbert et al. 2014b), developmental TCE exposure has also been associated with neurotoxicity including decreased learning and increased locomotor and exploratory activity in mice (Taylor et al. 1985; Isaacson et al. 1990; Blossom et al. 2013, 2016). One question arising from these results is the role of peripheral CD4^+^ T-cells in TCE-mediated developmental neurotoxicity. Studies by Blossom et al. (2013, 2016) have pointed to oxidative stress- and peripheral inflammation-driven altered glutathione metabolism in the cerebellum that may lead to DNA methylation deficits and subsequent behavioral alterations with developmental exposure. Thus, systemic effects may be contributing to TCE-induced developmental neurotoxicity via crosstalk between immune cells in the periphery and in the central nervous system (CNS). This crosstalk may occur indirectly via the production of inflammatory mediators in the periphery or directly through interaction of brain-infiltrating CD4^+^ T-cells with microglia, the resident macrophages of the CNS, and other cell types in the CNS. While both scenarios are plausible, studies to determine a causative role for CD4^+^ T-cells in mediating this neurotoxicity, either by using knockout mice or adoptive transfer experiments, have not been conducted.

Before this mechanism can be addressed, it is important to study additional neurotoxic endpoints that may be important in TCE-mediated neurotoxicity. While an extensive assessment of oxidative stress-related responses in the brain have been conducted with developmental TCE exposure in male mice (Blossom et al. 2008, 2013, 2016), the effects of TCE on microglia and key neuroinflammatory endpoints in the cerebellum in both sexes have not been conducted. To better understand responses in the CNS, this study was designed to explore and describe some of the basic markers of inflammation to begin to test a hypothesis that developmental TCE exposure may induce neurotoxicity through neuroimmune interactions.

## Materials and methods

### Animals

Adult male and female MRL^+/+^ mouse breeding pairs (6–8 wk-of-age) were purchased from Jackson Laboratories (Bar Harbor, ME). This strain of mouse spontaneously develops a mild lupus-like disease and is routinely used for studies of compounds, such as TCE, that induce autoimmune disease. In addition to immunotoxicity and autoimmunity, MRL^+/+^ mice have increased brain oxidative stress and redox imbalance and behavioral alterations with TCE exposure. This strain has been shown to exhibit a more aggressive and sustained microglial inflammatory response following mechanical injury. (Hampton et al. 2004). Thus, MRL^+/+^ mice were used in the current study to further examine neuroinflammatory responses.

### Experimental design

Continuous developmental exposure to TCE has been described (Blossom & Doss 2007). Briefly, gestational day 0 (GD0) female mice were divided (following stratified randomization) into two treatment groups and given ultrapure unchlorinated water with 0 (vehicle control) or 0.5 mg/mL TCE (purity >99%; Sigma, St. Louis, MO) as drinking water *ad libitum*. This dose of TCE has been used previously in studies of continuous developmental/early-life exposure (Blossom & Doss 2007). Drinking water also contained 1% Alkamuls EL-620, an emulsifier consisting of ethoxylated castor oil (Rhone-Poulenc, Cranbury, NJ), a reagent used to solubilize the TCE. Drinking water was changed three times/week to offset degradation of TCE. Dams produced 18 litters (nine control and nine treated) and all offspring remained with each dam until weaning. Maternal exposure to TCE or vehicle control containing drinking water continued throughout birth and lactation; once pups were weaned at post-natal day 21 (PND21), they were exposed to vehicle control or TCE directly in their drinking water for the duration of the experiment. All studies were approved by the Institutional Animal Care and Use Committee at the University of Arkansas for Medical Sciences.

At 49 days of age, mice were deeply anesthetized with inhaled isoflurane and exsanguinated and euthanized by decapitation. Brains from 2 to 4 randomly selected male and female offspring per litter were immediately removed and weighed. Brains were cut in half sagittally, and one half of each brain was immediately snap-frozen and stored at −80°C for later analysis of interleukin (IL)-6 and IL-10 concentrations. The other half was immersion-fixed for up to 24 h in 10% phosphate-buffered formalin and then transferred into 70% ethanol until processing for immunohistochemical analysis. Brains were not perfused with saline or fixative prior to euthanasia.

### Determination of IL-6 and IL-10 in brain tissue

Frozen brains were homogenized in a Tenbroek tissue homogenizer using tissue homogenization buffer (20 mM Tris-HCl (pH 7.4), 250 mM sucrose, 1 mM EDTA, 1 mM EGTA, 100× Halt Protease Inhibitor Single-Use Cocktail [EDTA-free]), and DEA (0.4% diethylamine, 100 mM NaCl). Homogenates were centrifuged at 14 000 rpm at 4 °C for 1 h and supernatant was collected. Total protein concentration in supernatants was measured via a standard Bradford protein assay. Supernatants were then assayed with ELISA kits for mouse IL-6 and mouse IL-10 (Abcam, Cambridge, MA). In brief, supernatants were diluted 5-fold and added in duplicate to 96-well plates pre-coated with IL-6 or IL-10 capture antibody. Plates included blank wells and standards for the creation of a standard curve. Subsequent steps included incubations with anti-mouse IL-6 or IL-10 detection antibody with biotin, horseradish peroxidase-streptavidin solution, and substrate reagent, and included washes between each step. After incubation with substrate reagent, stop solution was added to all wells, and plates were read at 450 nm in a Synergy HT plate reader (BioTek Instruments, Winooski, VT). A standard curve of absorbance and concentration was created from the values in the standard wells; concentration in samples was determined by extrapolation from the standard curve. Concentrations were adjusted to total protein content of supernatant for statistical analysis.

### Evaluation of CD3^+^ T-cells and microglia in brain tissue

Fixed brain halves stored in 70% ethanol were processed and embedded into paraffin blocks with the cerebella facing outward as this was the area of interest determined from previous studies (Blossom et al. 2008, 2013, 2016). Cerebella were sliced in 10-μm sections, and 16 sections/cerebella were mounted onto coated microscope slides (2–4 sections/slide). After de-paraffinization, epitope retrieval with citrate buffer, quenching of endogenous peroxidases, and blocking of nonspecific antigens, half of all slides/brain were incubated with anti-CD3^+^ (1:100; Abcam) to visualize T-cells or with anti-ionized calcium binding adaptor molecule 1 (Iba1; 1:500; Abcam) to visualize microglia. Appropriate secondary antibody was applied and 3,3-diaminobenzidine (DAB) with nickel solution was added for color development. Sections were counterstained with hematoxylin and cover-slipped. All parts of all 8 sections/brain stained with anti-CD3^+^ were viewed at 20× magnification; T-cell staining density was evaluated according to a scale where 0 = no stained T-cells; 1= a few stained T-cells; 2 = moderate number of cells; and 3 = substantial number of cells.

For sections stained with Iba1, the total number of microglia was counted at 20× magnification and assigned a morphology of either ameboid or ramified. As microglia can occupy many morphological states between ameboid or ramified, the cells were classified as ameboid if they had a large soma and projections that were short, stout, and/or absent and as ramified if they had a small soma and projections that were long and thin. The ratio of ameboid:total cells was calculated as the number of ameboid microglia divided by the total number of microglia counted.

### Statistical analysis

Data are presented as mean ± SD. Data were initially evaluated with a two-way analysis of variance (ANOVA) to determine potential interaction effects between treatment and sex. Two-way ANOVA did not reveal interaction effects, so pairwise comparisons within sex and between treatments and between sex within treatments were made with Student’s *t*-test and with an *F*-test for equality of variances. Statistical significance was determined at *p* values <0.05. In addition, effect sizes [(mean_control_−mean_treatment_)/standard deviation_control_)] were calculated to better distinguish trivial effects (<0.1) from large effects (>0.5).

## Results

### TCE exposure and characteristics of dams and offspring

Dams and their offspring were weighed weekly to obtain an average body weight and water consumption was monitored. As shown in Table 1, the amount of TCE (mg/kg/day) was based on average water intake, body weight, and a calculated average of ~20% TCE degradation in the water bottles. Based on this estimate, the mice given water containing 0.5 mg TCE/ml were exposed to TCE at levels of ≈145 mg/kg/day (via maternal exposure through gestation and lactation) and 61 and 68 mg/kg/day (male and female offspring, respectively) through direct exposure of the drinking water from PND20 until euthanasia at PND49. Although the exposure levels of TCE resulting from maternal exposure were not determined, the dose of TCE from direct exposure (PND21-PND49) were lower than the current 8-h Permissible Exposure Limit (PEL) established by the Occupational Safety and Health Administration (OSHA) for TCE of 100 ppm [or ≈76 mg/kg/day].

**Figure 1:**
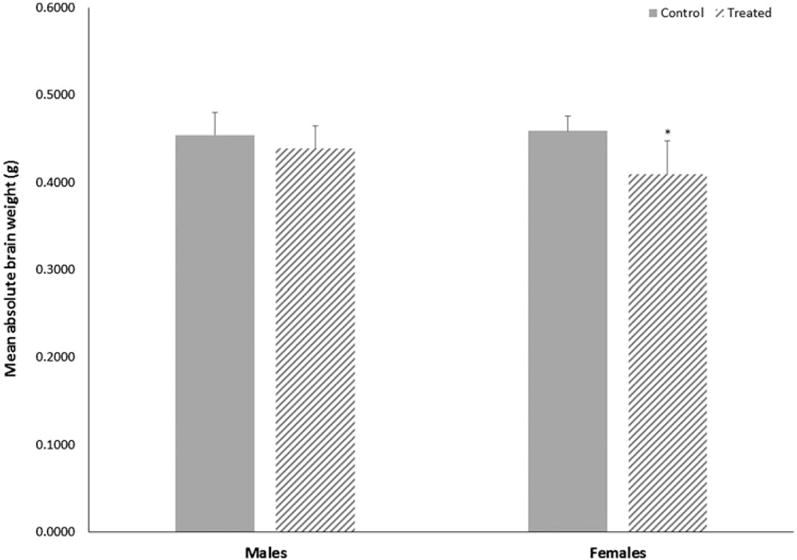
Terminal absolute brain weights of male or female MRL^+/+^ mice given TCE (treated) via drinking water from conception through 49 days-of-age. Values shown are means (g) ± SD. *Statistically significant difference in brain weight relative to control group of matching sex (*p* < 0.002).

While TCE exposure did not alter the weight of dams, it did alter mean weights of offspring when measured at study terminus (PND49). Both male and female offspring treated with TCE weighed less than control offspring, although the average weight of male offspring was not statistically reduced. Female offspring treated with TCE weighed 13% less relative to control offspring (*p* < 0.004 with a large effect size of 1.2) and male offspring treated with TCE weighed about 8% less relative to control offspring (*p* < 0.07 with a large effect size of 0.6). At study terminus, absolute brain weights of female mice treated with TCE were 11% lower (*p* < 0.002 with a large effect size of 3.0) relative to brain weights of control females (Figure 1). However, when adjusted by body weight, relative brain weight of female mice exposed to TCE did not differ statistically from relative brain weight of control female mice. Additionally, total protein content in brain supernatants did not differ statistically between control and treated females. No statistical differences were observed in brain weights or total protein content of male mice.

### IL-6 and IL-10 in brain tissue

**Figure 2:**
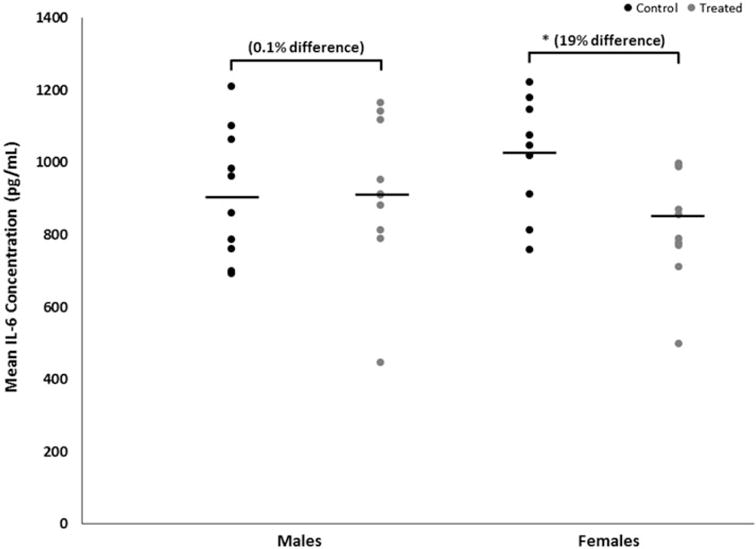
Brain-specific concentrations of IL-6 of male or female MRL^+/+^ mice given TCE (treated) via drinking water from conception through 49 days-of-age. Values shown are means (pg/ml) ± SD. Statistically significant difference in IL-6 concentrations relative to control group of matching sex (*p* < 0.05).

**Figure 3:**
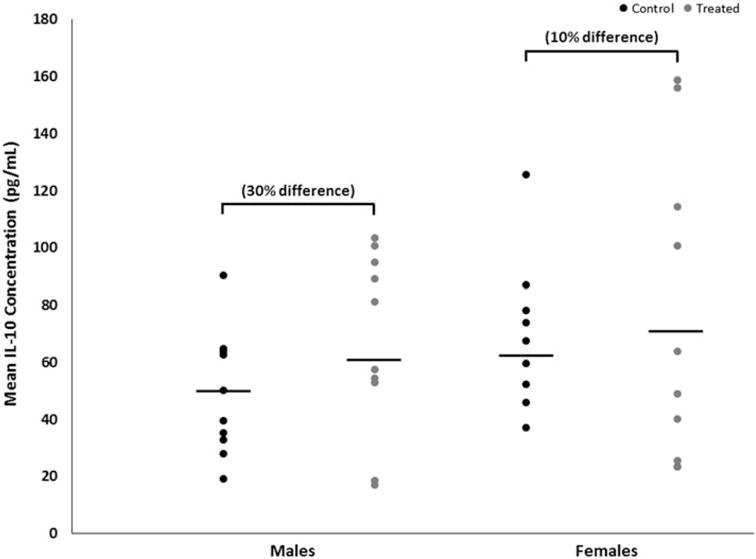
Brain-specific concentrations of IL-10 of male or female MRL^+/+^ mice given TCE (treated) via drinking water from conception through 49 days-of-age. Values shown are means (pg/ml) ± SD.

Mean brain concentrations of IL-6, with and without adjustment for total brain protein content, were decreased by 19% (*p* < 0.02 with a large effect size of 1.2) in female mice developmentally exposed to TCE relative to concentrations in control females (Figure 2). In exposed male mice, brain IL-6 concentrations were not altered by treatment (*p* < 0.1 with a trivial effect size of 0.06). IL-6 concentration did not differ between the sexes within either treatment. Conversely, brain IL-10 concentrations were increased by developmental TCE exposure (Figure 3), but not statistically. Relative to concentrations in brains from the control group, with and without adjustment for total brain protein content, mean brain concentrations of IL-10 were increased by about 30% (*p* < 0.3 with a large effect size of 0.6) in exposed male mice and by about 10% (*p* < 0.7 with a moderate effect size of 0.3) in exposed female mice. IL-10 did not differ between sexes within either treatment.

### CD3^+^ T-cells and microglia in brain tissue

**Figure 4:**
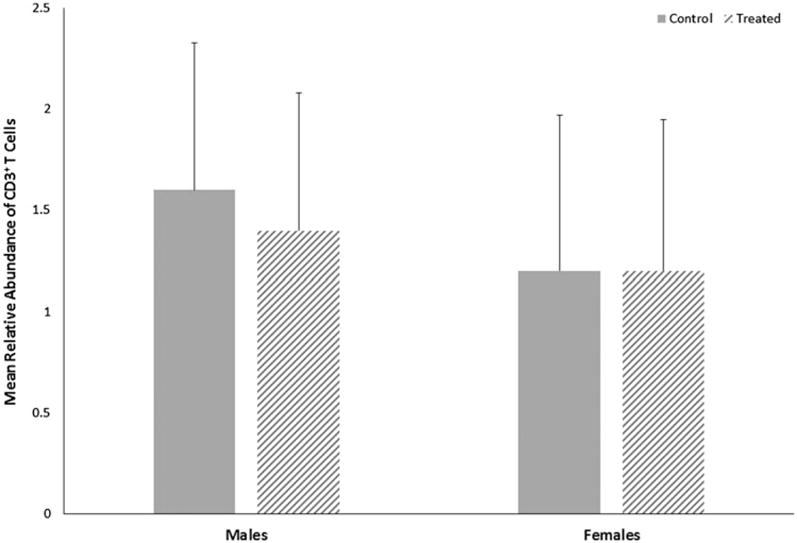
Relative abundance of CD3^+^ T-cells in cerebella of male or female MRL^+/+^ mice given TCE (treated) via drinking water from conception through 49 days-of-age. Values shown are means ± SD.

**Figures 5:**
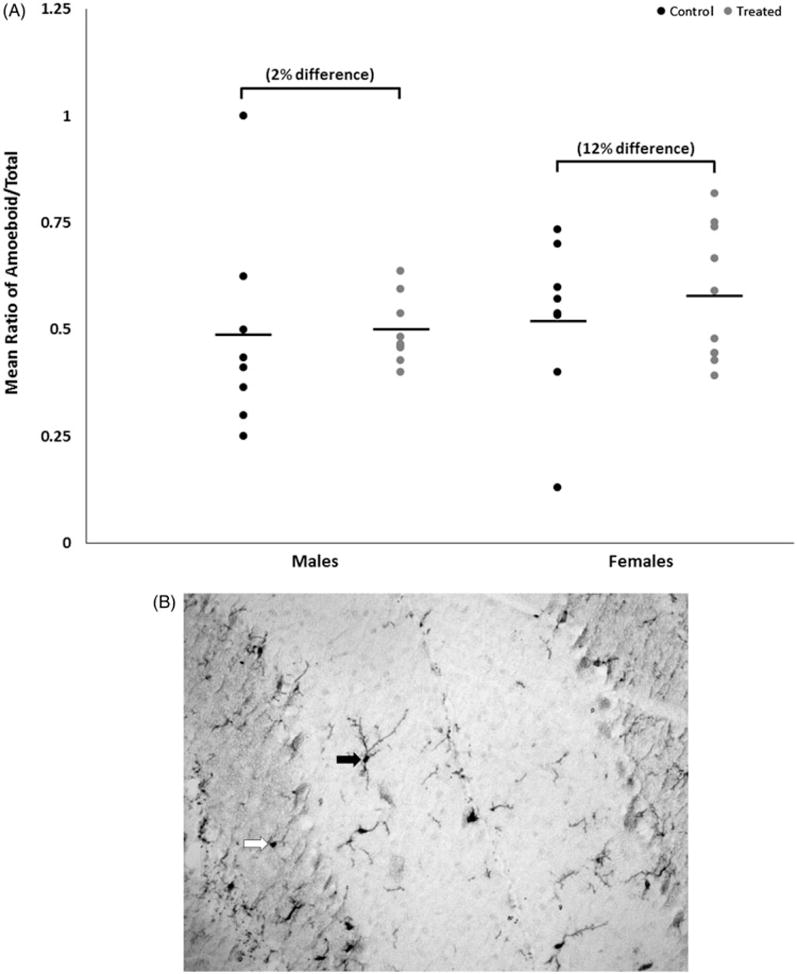
(A) Representative image of microglial staining. 20× magnification. Solid arrow: microglia classified as ramified. Open arrow: microglia classified as ameboid. (B) Mean ratio of microglia with ameboid morphology to total number of microglia (means ± SD) in cerebella of male or female MRL^+/+^ mice given TCE (treated) via drinking water from conception through 49 days-of-age.

Both control and treated groups of female mice had slightly lower CD3^+^ T-cell abundance relative to male mice (Figure 4); however, the relative abundance of CD3^+^ T-cells in sections of cerebellar tissue did not differ statistically within sex by treatment or between sexes within treatment. Overall, the ratio of the number of ameboid microglia to the total number of microglia (ameboid + ramified) in cerebellar tissue did not differ statistically within sex by treatment or between sexes within treatment (Figures 5(A,B)), although treated female mice had slightly more ameboid microglia relative to control female mice, with a moderate effect size of 0.3.

## Discussion

Developmental exposure to TCE has been associated with both immunotoxicity and neurotoxicity in an MRL^+/+^ mouse model as well as in humans occupationally exposed to relatively high levels of TCE. Immunotoxicity presents as expansion of activated/memory CD4^+^ T-cells with a T-helper (T_H_)-1-like pro-inflammatory phenotype in the MRL^+/+^ mouse model (Blossom & Doss 2007; Blossom et al. 2008; Gilbert et al. 2014b). A study with B_6_C_3_F_1_ mice, a strain of mouse not prone to autoimmune disease, also demonstrated alterations in T-cell subpopulations as well as suppression of the T-cell-dependent antibody response and increases in the delayed-type hypersensitivity response (Peden-Adams et al. 2006). Developmental neurotoxicity also arises from TCE exposure, resulting in altered redox homeostasis and a decrease in neuroprotective factors such as brain-derived neurotrophic factor (Blossom et al. 2012), and an increase in oxidative stress in specific brain regions (Blossom et al. 2016). Due to the presence of both developmental immunotoxicity and neurotoxicity, we hypothesized that neuroimmune biomarkers in the brain following continuous exposure to TCE from conception through adult-hood would be altered. As little mechanistic data exist for TCE-induced neurotoxicity, these data begin to address a major knowledge gap on the linkages between the developing immune and nervous systems following developmental TCE exposure.

The most obvious cell linking the immune and nervous systems is the microglia, the resident macrophage of the CNS. Microglia play key role in shaping and maintaining the CNS throughout life. As a result of their microenvironment, microglia have developed numerous response pathways to ensure that collateral damage in highly sensitive tissues does not result from potentially neurotoxic immune activation (Matcovitch-Natan et al. 2016). These response pathways are formed during development and can arise from signals originating inside and outside the CNS. Matcovitch-Natan et al. (2016) recently identified at least three distinct phases of regulatory network formation in microglia that can lead to functional effects when perturbed. While several studies have raised questions about the role of microglia in specific neurodevelopmental disorders such as Autism Spectrum Disorder, Rhett Syndrome, and schizophrenia (Maezawa et al. 2011; Hsiao 2013; Takahashi & Sakurai 2013) and a few have evaluated glial cell responses in fetal alcohol syndrome (Guizzetti et al. 2014; Wilhelm & Guizzett 2016), only a handful of studies specifically address how microglia respond to toxicants. Many of these studies utilize adult models and typically focus on signals thought to arise from M1, or classic-ally activated, pro-inflammatory microglia. For example, O’Callaghan et al. (2008) described that following neurotoxic insults (i.e. drugs, chemicals, trauma, and disease), damage signals or the insults themselves activate microglia, which may lead to toxicity in neural targets. While this is certainly one possible outcome following an exogenous insult, other consequences may result, especially when the insult occurs during development. As, if Appel et al. (2010) asserts, the balance of phenotypes is important for maintaining neuroprotection, this is likely critically important during neurological development, when synapses are being formed, shaped, and pruned. Insults that disrupt this fine balance may produce a spectrum of effects, from profound dysfunction to subtle changes in susceptibility to subsequent life events.

Peripheral and infiltrating CD4^+^ T-cells are thought to interact with microglia in a highly complex manner, resulting in phenotypic changes in microglia and in T-cells. For example, T-cells may dictate whether microglia become pro-inflammatory (an M1 activated phenotype) or anti-inflammatory (an M2 activated phenotype) or conversely, M1 or M2 activated microglia may promote the proliferation and function of T-cells into different functional lineages (Appel et al. 2010). While it is unclear which cell type dictates the ultimate phenotype in a particular situation, it is clear that the balance of phenotypes is important for maintaining neuroprotection (Appel et al. 2010). When the balance is skewed, in the case of neurodegenerative diseases, for example, it appears as if M1 activated microglia predominate, leading to neuron damage by cytotoxic actions of T-cells (Appel et al. 2010). Whether this situation is similar in a developing CNS and in other diseases of the CNS is unknown. Therefore, it is possible that developmental TCE exposure, through expansion of CD4^+^ T-cells in the periphery, skews microglia toward a phenotype that is more toxic than protective during development. Current studies of microglia in vivo are establishing a diversity of phenotypes that diverges from this traditional M1/M2 dichotomy; it is likely that microglia phenotype includes a spectrum of somewhat different and overlapping functional phenotype subpopulations (Hu et al. 2015). These different sub-populations are thought to have unique physiological features and specific biological functions, but in situations of CNS injury, M1/M2 polarization is thought to occur (Hu et al. 2015). Developmental TCE exposure may therefore polarize microglia toward a phenotype that promotes neuroinflammation.

In the current study, MRL^+/+^ mice were exposed to a single dose of TCE from conception through adulthood. This exogenous insult was present during every major developmental milestone. To begin to formulate a profile of neuroinflammation associated with developmental TCE exposure and to understand the role that microglia might play in TCE-induced neurotoxicity, we evaluated several markers relevant to neuroinflammation and microglia: brain levels of IL-6 and IL-10, T-cell staining, and microglial morphology. While the results here reflect a one-time snapshot the brain after nearly 70 days of continuous TCE exposure at a single dose, one can begin to speculate about the neuroinflammatory profile of these animals. In comparison to control animals, developmental TCE exposure in female animals reduced absolute brain weights and levels of IL-6 and increased levels of IL-10 and the relative number of ameboid microglia.

While only changes in absolute brain weights and levels of IL-6 were statistically significant and produced large effect sizes in female animals, changes in levels of IL-10 and the relative number of ameboid microglia between control and treated female animals produced moderate effect sizes. We hypothesize these changes are biologically relevant and reflect recovery or compensation from the neuroinflammatory effects induced by developmental TCE exposure. Blossom et al. (2016) demonstrated that the developmental exposure to TCE that expands the effector/memory subsets of peripheral CD4^+^ T-cells also increases their production of pro-inflammatory cytokines, inflammatory biomarkers in the plasma, and cerebellar redox homeostasis and that these changes accompanied neurotoxicity. Additionally, Blossom et al. (2012) noted a reduction in hippocampal mRNA expression levels of key neurotrophic factors (i.e. BDNF, NGF, NT-3), which they suggested was a mechanistic link to increased oxidative stress. The loss of neurotrophic support with neuroinflammation and oxidative stress has been linked with human neurodevelopmental disorders (Sajdel-Sulkowska et al. 2011). However, very little mechanistic data exist to explain why developmental TCE exposure leads to neurotoxicity. In a study with prenatal only exposure to TCE, changes in male offspring behavior were accompanied by evidence of immunomodulation and inflammation, including increases in central memory CD4^+^ T-cells and their production of pro-inflammatory cytokines (Blossom et al. 2016). The authors hypothesized that neurotoxicity could have arisen from the activation of these peripheral CD4^+^ T cells, leading to oxidative stress, neuroinflammation, and ultimately, changes in behavior.

Several toxicants have been associated with brain hypoplasia when exposure occurs during development, notably methyl mercury, ethanol, styrene, xylene, and also induce significant changes in brain function and behavior (Koger et al. 2005). Brain hypoplasia can arise from myriad mechanisms and is a well-known pathology of organic solvent exposure and abuse, including exposure and abuse during pregnancy. However, brain hypoplasia following TCE exposure is not a commonly reported outcome in occupationally exposed humans or in experi-mental animal models and when adjusted for body weight, this difference was no longer statistically significant. Additionally, while increased levels of brain IL-6 have been associated with neuroanatomical abnormalities in murine models (Smith et al. 2007; Wei et al. 2012), decreased levels are more difficult to explain. We hypothesize the decrease in IL-6 combined with the mild increase in IL-10 reflects reestablishment of the anti- vs. pro-inflammatory cytokine balance that was skewed toward a pro-inflammatory profile by developmental TCE exposure. In male animals, the increase in IL-10 was more pronounced (30% difference between control and treated animals with a large effect size) and the decrease in IL-6 was trivial. This is further supported by evidence from studies of TCE exposure in macrophages in which a decline in IL-6 over 40 weeks of exposure was observed (Gilbert et al. 2014a). Interestingly, in that study, TCE liver pathology correlated with a loss of hepatic IL-6 R and gp130, the components of the IL-6 receptor. These results suggested that a loss of IL-6 signaling in the liver would be expected to exacerbate inflammation (Gilbert et al. 2014a). These results also implied that TCE regulates IL-6 and IL-6 signaling in female mice, which is consistent to results in the female mice presented here.

Based on these previous findings in the liver and in macrophages, we assert that the brain hypoplasia in female mice was associated with alterations in IL-6 signaling that likely occurred during critical periods of brain development. The moderate increase in the number of ameboid microglia to ramified microglia could reflect a shift in the phenotypic profile of microglia in response to changes in IL-6. However, additional studies are necessary to fully describe the phenotypic/neuroinflammatory profile after developmental TCE exposure. These studies will require evaluation of brains from birth through adulthood that includes descriptions of microglia phenotype and measurements of pro- and anti-inflammatory cytokines from various cell types. In addition to a more comprehensive assessment of microglia phenotype at different ages, experiments to evaluate the dose-response of TCE will be conducted to better understand the neuroinflammatory profile associated with TCE exposure. Mechanistic studies will assess *ex vivo* responses of microglia to the expanded CD4^+^ T-cell population to determine how these cells are influencing one another and the sex-differences associated with this relationship.

## Conclusions

MRL^+/+^ mice exposed to TCE from conception through early adulthood failed to develop robust changes in neuroinflammatory markers. However, moderate to large changes observed in pro- and anti-inflammatory cytokines coupled with changes in microglial morphology are suggestive of the emergence of an anti-inflammatory/neuroprotective phenotype. It is possible, based on previous findings of the effects of TCE exposure on liver and on macrophages, that this is a compensatory response to alterations in IL-6 signaling induced by developmental TCE exposure. To fully appreciate the dynamics of developing and interacting systems, additional work is planned to assess microglial responses to cytokine signals from CD4^+^ T-cells across the developmental continuum.

## Figures and Tables

**Table 1:** Water consumption and body weights (BW) of MRL^+/+^ dams and offspring exposed to vehicle (control) or trichloroethylene (TCE) via drinking water from conception through adulthood at post-natal day (PND) 49.

	Control dam (*n* = 9)	TCE dam (*n* = 9)	Control male (*n* = 18)	TCE male (*n* = 20)	Control female (*n* = 18)	TCE female (*n* = 20)
Mean dam BW (g), PND21-49	38.95 (1.87)	38.84 (1.98)	34.30 (3.89)	27.21 (4.65)	27.50 (4.47)	24.52 (3.18)
TCE consumption (mg/kg/day)	0	145.55 (28.52)	0	61.76 (11.04)	0	68.83 (8.94)
Offspring BW at terminus (g)	–	–	34.28 (4.45)	31.65 (4.25)	30.73 (3.35)	26.74 (4.46)^a^

Values shown are means (SD in parentheses).

a Statistically different from BW of sex-matched control group (*p* < 0.05).
